# Androgen and Anti-Mullerian Hormone Concentrations at Term in Newborns and Their Mothers with and without Polycystic Ovary Syndrome

**DOI:** 10.3390/jcm8111817

**Published:** 2019-11-01

**Authors:** Martina Kollmann, Barbara Obermayer-Pietsch, Elisabeth Lerchbaum, Uwe Lang, Sereina A. Herzog, Christian Trummer, Anna Scheuchenegger, Daniela Ulrich, Philipp Klaritsch

**Affiliations:** 1 Division of Obstetrics and Maternal Fetal Medicine, Department of Obstetrics and Gynecology, Medical University of Graz, 8036 Graz, Austria; uwe.lang@medunigraz.at (U.L.); daniela.ulrich@medunigraz.at (D.U.); philipp.klaritsch@medunigraz.at (P.K.); 2 Department of Internal Medicine, Division of Endocrinology and Diabetology, Medical University of Graz, 8036 Graz, Austria; barbara.obermayer@medunigraz.at (B.O.-P.); elisabeth.lerchbaum@medunigraz.at (E.L.); christian.trummer@medunigraz.at (C.T.); 3 Institute for Medical Informatics, Statistics and Documentation (IMI), Medical University of Graz, 8036 Graz, Austria; herzog.sereina@gmail.com; 4 Centre for Health Economics Research and Modelling Infectious Diseases (CHERMID), Vaccine and Infectious Disease Institute (VAXINFECTIO), University of Antwerp, Prinsstraat 13 2000 Antwerp, Belgium; 5 Division of Neonatology, Department of Pediatrics and Adolescence Medicine, Medical University of Graz, 8036 Graz, Austria; a.scheuchenegger@medunigraz.at

**Keywords:** polycystic ovary syndrome, PCOS, androgen concentration, PCOS offspring

## Abstract

**Objectives:** The aetiology of polycystic ovary syndrome (PCOS) is not particularly mapped; however, a complex interaction of various factors, such as genetic, environmental and intrauterine factors, can be assumed. Experimental animal studies and clinical observations support the hypothesis that developmental programming by excess intrauterine steroid is relevant. The aim of the study was to investigate whether mothers with and without PCOS exhibit different androgen and anti-Mullerian hormone (AMH) levels at the end of pregnancy and how maternal hormone levels are reflected in their offspring. **Methods:** Between March 2013 and December 2015, we performed a prospective cross-sectional study at the Medical University of Graz. We included 79 women with PCOS according to the ESHRE/ASRM 2003 definition and 354 women without PCOS, both with an ongoing pregnancy ≥37 + 0 weeks of gestation, who gave birth in our institution. Primary outcome parameters were the levels of maternal and neonatal androgens (testosterone, free testosterone, androstenedione) and AMH at delivery. **Results:** Androgen levels in female offspring of PCOS and non-PCOS women at birth did not differ, while maternal hormone levels differed significantly. Androgen levels in PCOS boys were significantly higher when compared to levels in PCOS girls. **Discussion:** Our findings do not support the hypothesis that maternal androgen excess contributes to elevated androgen concentrations in the female offspring. Nevertheless, the effects of the increased androgen concentrations in mothers on their offspring have to be investigated in future studies.

## 1. Introduction

Polycystic ovary syndrome (PCOS) is a heterogeneous endocrine disorder which affects various body systems and leads to reproductive and metabolic complications [1,2,3,4]. The prevalence of PCOS varies depending on ethnicity, body composition and the definition used for diagnosis [5,6,7,8,9,10]. It is found in 5–10% of women of reproductive age and in up to 30% of the subgroup with overweight and obesity [9,10]. The aetiology of PCOS is not particularly mapped; however, a complex interaction of various factors, such as genetic, environmental and intrauterine ones, can be assumed [11]. Evidence for a genetic basis of PCOS was already reported in 1968 [12]. With the performance of genome-wide association studies (GWAS) new insights into heritability of the syndrome were gained [13,14]. Epigenetic variations, including gene methylation, histone modification, microRNAs and RNA binding proteins may also play a role in determining the PCOS phenotype [15,16,17,18]. Environmental determinants of PCOS, such as environmental toxins, diet and nutrition, socioeconomic status and geography were summarized in a recently published review [19]. The third factor which seems to play a crucial role in the development of PCOS is the intrauterine milieu during pregnancy and early childhood [11,20,21,22]. Experimental animal studies and clinical observations support the hypothesis that developmental programming by steroid excess is relevant [23,24,25,26,27]. Sex differences in prenatal androgen levels have been detected, and testosterone (T) levels in umbilical cord blood and in amniotic fluid are higher in healthy male babies than in healthy female babies [28,29]. There are only a few studies reporting on the relationship between maternal androgen and anti-Mullerian hormone (AMH) levels during pregnancy and between androgen and AMH levels in the respective offspring in PCOS women, with inconsistent results [30,31,32,33,34,35,36]. The aims of the current study were firstly, to investigate whether mothers with and without PCOS have different androgen and AMH levels and secondly, how maternal hormone levels are reflected in their offspring at birth.

## 2. Materials and Methods

### 2.1. Study Design

This prospective cohort study was performed at a single academic tertiary hospital (Department of Obstetrics and Gynaecology, Medical University of Graz, Graz, Austria) between March 2013 and December 2015.

### 2.2. Ethical Approval

The study was approved by the institutional review board (Ethics committee at the Medical University of Graz, Graz, Austria; 24-179ex11/12). Participants provided their written informed consent to participate in this study.

### 2.3. Participants

Women with PCOS according to the ESHRE/ASRM 2003 definition [7] and an ongoing pregnancy ≥37 + 0 weeks of gestation and women without PCOS and an ongoing pregnancy ≥37 + 0 weeks of gestation were invited to participate. PCOS was diagnosed following a clinical and sonographic evaluation and a hormonal analysis before pregnancy. Ultrasound examinations were performed by medical doctors specialized in obstetrics and gynaecology. Clinical investigations and hormonal analyses were routinely performed in cooperation with the Division of Endocrinology and Diabetology at the Department of Internal Medicine at Medical University of Graz, Austria. We report on a population of women who were treated in our specialized unit and delivered at our institution. Only singleton pregnancies were included. Patients with severe comorbidities (neurodegenerative disease, immune mediated disease, cardiovascular disease, infectious disease), suspected abnormal placentation (placenta accreta, increta or percreta), placenta previa, previous vertical uterine incision, a history of major abdominal surgery, or known foetal malformations were excluded. Eligible patients were approached by members of the research team, and written informed consent was obtained from all participants.

### 2.4. Outcome Measures

Primary outcome parameters were the levels of maternal and neonatal androgens (T, free testosterone (fT), androstenedione (ANDR)) and AMH at delivery.

### 2.5. Data Sources/Measurement

Blood samples were collected from mothers and neonates within the first 5 min after delivery. The neonatal sample comprised mixed umbilical cord blood. Laboratory kits and assays did not change between 2013 and 2015 for T and ANDR. For AMH, the assay was changed in November 2014 from ultra-sensitive anti-Müllerian hormone/Müllerian-inhibiting substance enzyme-linked immunosorbent assay (ELISA) kit (Ansh Labs, Webster, TX, USA) to Access 2 immunosorbent assay system (Beckmann Coulter, Brea, CA, USA). Assays were compared and showed good correlation (r = 0.95). T and sexual hormone-binding globulin (SHBG) levels were measured on a daily basis via radioimmunoassays (RIA) or ELISA, respectively, and were stored at 4 °C until analysis. ANDR and AMH levels were measured on a weekly basis, and blood samples were frozen and stored at −40 °C until analysis. fT was measured on a daily basis via RIA and confirmed with a second method [37]. Demographic data were extracted from the local perinatal database (PIA, ViewPoint, GE Healthcare, Solingen, Germany) and the medical documentation system or patient files. Pre-pregnancy body weight and height were used to calculate the body mass index (BMI). Information on current smoking status was collected at admission.

### 2.6. Sample Size

To detect a significant difference in T with an alpha of 0.05 and a power of 80% for an effect size of 0.5, we planned to recruit at least 35 patients with PCOS and 350 non-PCOS patients. As we expected some drop-outs and difficulties with cord blood analysis, we aimed to include at least 400 patients. A data quality check after one year of recruitment revealed that more patients than expected had to be excluded due to comorbidities and that cord blood analysis was not feasible in some cases due to insufficient material. We therefore aimed to recruit at least 80 PCOS patients and 420 non-PCOS patients.

### 2.7. Statistical Methods

For categorical variables, relative and absolute proportions are indicated, and continuous variables are expressed as mean ± standard deviation or median with range, respectively. Categorical variables were analysed by using Fisher’s exact test or chi-square test, while continuous outcomes comparison between two groups were done using *t* test or Mann-Whitney U-test. To assess the association between maternal and neonatal hormone levels, Spearman’ rho correlation coefficients were calculated. All analyses were performed using the statistic software R (version 3.3.3, Vienna, Austria) [38]. A *p*-value < 0.05 was considered to be statistically significant.

## 3. Results

### 3.1. Participants

A total of 499 pregnant women were assessed for eligibility, and 433 were eventually included for analysis (79 with PCOS according to ESHRE/ASRM 2003 definition and 354 non-PCOS women). Four women declined to participate, 7 women were excluded due to twin pregnancies (4 PCOS, 3 non-PCOS), and 55 patients (11 PCOS, 44 non-PCOS) were excluded due to severe maternal or foetal comorbidities.

**Figure 1:**
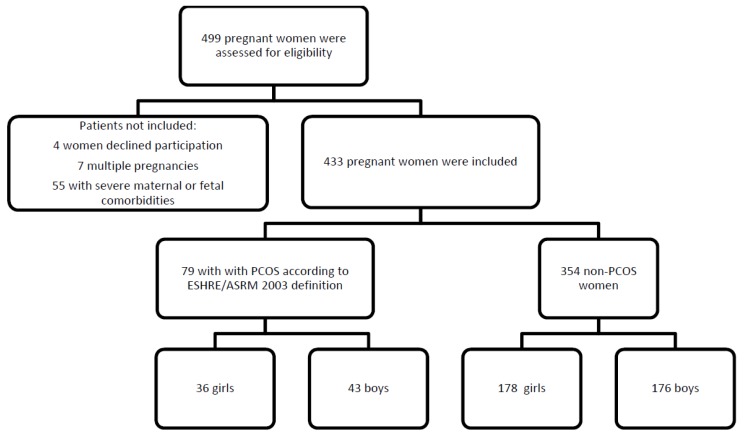
Flow chart presenting the study population and distribution. PCOS: polycystic ovary syndrome.

PCOS women gave birth to 36 (45.6%) girls and 43 (54.4%) boys. In the non-PCOS group, 178 (50.3%) girls and 176 (49.7%) boys were born (Figure 1).

### 3.2. Descriptive Data

Maternal age, BMI, gestational age at delivery and the proportion of smokers were comparable (Table 1). Before pregnancy, 46 (58.2%) PCOS women presented with hyperandrogenaemia, 67 (84.8%) with clinical hyperandrogenism, 53 (67.1%) had polycystic ovaries, and 68 (86.1%) presented with oligo-amenorrhoea. Forty-one (51.9%) women in the PCOS group and 27 (7.6%) in the control group reported that they did not conceive within one year. Regarding the actual pregnancy, 67 (84.8%) in the PCOS group versus 346 (97.7%) in the control group conceived spontaneously; 7 (8.9%) versus 1 (0.3%) became pregnant after stimulation and ovulation induction, 5 (6.3%) versus 7 (1.9%) were treated with assisted reproductive technology (ART).

### 3.3. Testosterone

The mean maternal T levels were 1.17 ng/mL (range 0.44–4.23) in PCOS women and 0.97 ng/mL (0.18–5.56) in non-PCOS women. The levels were significantly higher in PCOS women (*p* < 0.001). The mean T levels were 1.54 ng/mL (0.84–5.82) in PCOS girls and 1.82 ng/mL (0.88–13.05) in non-PCOS girls. These levels did not differ significantly (*p* = 0.230). The mean T levels in PCOS boys (2.17 ng/mL (1.16–10.10)) were comparable to the mean T levels in non-PCOS boys (1.76 ng/mL (0.95–9.52)). The mean T levels in PCOS boys were significant higher when compared to the mean T levels in PCOS girls (*p* = 0.021) (Table 2 and Table 3).

### 3.4. Free Testosterone

The mean maternal fT levels were 6.79 pg/mL (1.37–26.80) in PCOS women and 6.72 pg/mL (0.48–27.91) in non-PCOS women. The levels did not differ significantly (*p* = 0.563). The mean fT levels were 22.66 pg/mL (10.52–47.0) in PCOS girls and 23.89 pg/mL (6.12–72.01) in non-PCOS girls. These levels did not differ significantly (*p* = 0.196). The mean fT levels in PCOS boys (29.94 pg/mL (6.39–61.41)) were comparable to the mean fT levels in non-PCOS boys (24.08 pg/mL (5.23–73.07)). The mean fT levels in PCOS boys were significantly higher when compared to the mean fT levels in PCOS girls (*p* = 0.012) (Table 1 and Table 3).

### 3.5. Androstenedione

Maternal ANDR levels were 3.44 ng/mL (1.06–10) in PCOS women and 2.74 ng/mL (0.49–10) in non-PCOS women. The levels did differ significantly (*p* = 0.002). The mean ANDR levels were 2.19 ng/mL (1.08–7.77) in PCOS girls and 2.78 ng/mL (0.83–8.06) in non-PCOS girls. These levels did not differ significantly (*p* = 0.113). The mean ANDR levels in PCOS boys (3.47 ng/mL (1.26–7.93)) were significantly higher when compared to the mean ANDR levels in non-PCOS boys (2.29 ng/mL (0.83–9.24); *p* = 0.039) and in PCOS girls (*p* = 0.018) (Table 2 and Table 3).

### 3.6. AMH

Maternal AMH levels were 1.10 ng/mL (0.10–25.0) in PCOS women and 0.72 ng/mL (0.02–49.0) in non-PCOS women. The levels did differ significantly (*p* = 0.001). The mean AMH levels were 0.2 ng/mL (0.0–9.2) in PCOS girls and 0.2 ng/mL (0.0–25.0) in non-PCOS girls. The levels did not differ significantly (*p* = 0.975). The mean AMH levels in PCOS boys (22.0 ng/mL (14.4–45.6)) were comparable to the mean AMH levels in non-PCOS boys (20.01 ng/mL (1.6–124.2)). The mean AMH levels in PCOS boys were significantly higher when compared to the mean AMH levels in PCOS girls (*p* < 0.001) (Table 2 and Table 3).

The correlations between maternal and neonatal T levels (r = 0.33, *p* < 0.001), fT levels (r = 0.37, *p* < 0.001), ANDR levels (r = 0.42, *p* < 0.001) and AMH levels (r = 0.21, *p* < 0.001) were low.

## 4. Discussion

### 4.1. Key Results

The androgen levels in female newborns of PCOS and non-PCOS women at birth did not differ, while the maternal hormone levels differed significantly. The androgen levels of male newborns of PCOS women were significantly higher when compared to the levels of their female counterparts. The correlations between maternal and neonatal hormone levels were low.

### 4.2. Interpretation

Our findings do not support the hypothesis that elevated androgen concentrations in PCOS mothers lead to elevated levels in the offspring, at least when measured at birth. This hypothesis has been strongly encouraged by Abbott et al., who performed studies in rhesus monkeys, which were prenatally exposed to androgens [24,25,39,40,41,42,43,44]. So far, there are just a few human studies available dealing with this subject. In a recent study, researcher investigated androgen and AMH levels in 20 PCOS women and 83 controls at 20 weeks of gestation and at delivery and found similar results to ours [32]. Data on umbilical cord AMH levels in PCOS offspring are scarce, and the available studies so far showed inconsistent results [30,32]. Our study results are in agreement with the study by Caanen et al. and show, as expected, higher AMH levels in boys compared to girls. However, the levels in PCOS girls and non-PCOS girls were similar [32]. Another study by Anderson et al. included 39 PCOS patients and 31 controls [32]. They investigated mixed cord blood T, ANDR, dehydroepiandrosterone (DHEA), 17-hydroxyprogesterone, oestradiol (E2) and dihydrotestosterone (DHT) levels. T and DHEA did not differ significantly in the offspring [33]. Interestingly, the study found lower levels of ANDR in the cord blood of female offspring of PCOS women in comparison to female offspring of non-PCOS women, which was also confirmed by two other studies [32,33,36]. Cord blood E2 levels were also significantly lower in PCOS women, without any difference in the testosterone-to-oestradiol ratio. The authors suggest a decreased foetal or placental production of steroids [33]. Boutzios et al. performed a study on PCOS patients (*n* = 41), patients with gestational diabetes (*n* = 54), controls (*n* = 56) and the respective offspring [34]. The T levels did differ in the mothers but did not reveal any difference in the offspring [34]. Another study which was performed by Maliqueo et al. examined 20 pregnant PCOS women and 30 controls and showed similar results [36]. So far, only one study reported elevated T levels in the cord blood of female offspring of PCOS mothers compared to controls [41]. Overall, it seems that the hypothesis that maternal androgen excess contributes to elevated androgen concentrations in the female offspring and therefore leads to the development of PCOS cannot be supported, at least when androgen levels are measured at birth. However, there are no realistic causes for the situation to change during pregnancy.

Our finding may be explained by the existence of protective mechanisms which work throughout pregnancy [45,46,47]. First of all, the placenta forms an effective barrier by producing placental aromatase, which quickly catalyses the conversion of androgens [48]. Another mechanism is the increase of progesterone, which competes for androgen receptor binding and has an affinity for 5a-reductase, which further results in the inhibition of the conversion of T to the more potent DHT [49,50,51,52]. A further protective factor is the physiological increase of maternal SHBG, which leads to a higher share of bounded and biologically inactive sex steroids.

Although we did not find higher androgen levels in the offspring, a negative effect of the higher androgen concentrations in PCOS mothers during pregnancy cannot be ruled out. Altered maternal hormone levels might have their effect on PCOS offspring in a more indirect way, such as foetal programming. A study on zebrafish embryos which were exposed to androgens (T and DHT) showed altered global methylation levels in the ovary and elevated postprandial glucose levels [53]. Similar results were found by Zhang et al. and Xu et al., who examined the effect of a hyperandrogenic milieu in utero on rats [54] and on rhesus monkeys [16]. Data on humans are scarce. The first pilot study on PCOS patients and controls did not reveal a significant difference in global methylation (6.7% for PCOS women and 7.1% for controls) [18], whereas a more recent study reported a significant difference [17]. The latter study also looked at seven potentially interesting gene *loci* and found hypermethylation in the promotor region of *SLC2A8*, *NRIP1*, *IGF2BP2*, *AMHR2* and hypomethylation of *INSR* und *AMH* [17].

### 4.3. Limitations

A better way to examine hormone exposure during prenatal life would be the measurement of circulating foetal hormone levels at repeated time points during pregnancy. However, this would require cordocentesis and is therefore not without significant risk to the foetus; hence, surrogate markers of foetal hormone levels must be applied. A relatively simple way is the collection of umbilical cord blood at birth [55]. Alternatively, amniotic fluid samples could be investigated. However, this approach is also associated with a higher complication rate and, therefore, cannot be used in study settings. Furthermore, the exact correlation between hormone levels in foetal blood and amniotic fluid is not precisely known [55]. Foetal blood, which is loaded with placental steroid metabolites and some maternal steroids, leaves the placenta via the umbilical vein and returns from the foetus to the placenta via the umbilical arteries [56]. A review published in 2014 examined the accuracy and biological interpretation of the measurement of androgens and oestrogens in cord blood [55]. Usually, venous and arterial blood samples are taken at once, and the exact proportion of each component is not known precisely. Nonetheless, in spite of different steroid concentrations in umbilical arteries and vein, Pašková et al. showed that the concentrations strongly correlated [55,57]. However, one has to be cautious when interpreting hormone levels which were measured in umbilical cord blood. Various factors, such as obstetric and maternal ones, can influence the concentrations, although the extent of this influence is not exactly known [55]. On the one hand, it is known that gestational age and labour have an effect on foetal adrenal steroid production. Further, we know that the levels of steroid-metabolizing enzymes in the placenta are modulated by factors connected to labour and delivery, such as glucocorticoids, pro-inflammatory cytokines, and exposure to reactive oxygen species [55,58]. Recently published studies demonstrated that gestational age and delivery significantly influence androgen and oestrogen concentrations in cord blood [59,60]. Labour was associated with significantly lower median cord blood T and fT levels and higher SHBG, ANDR and DHEA levels. Gestational age at delivery was significantly negatively correlated with T and fT levels and significantly positive correlated with SHBG, ANDR and DHEA levels. The effect of an antenatal glucocorticoid administration was further investigated, and a significant effect was found [60]. We could not confirm these findings in our cohort of PCOS and non-PCOS women. None of our patients received glucocorticoid for lung maturation or other medical reasons. However, there was a significant higher rate of gestational diabetes in PCOS women, and a possible association should be investigated in further studies.

Another fact we have to bear in mind is that all PCOS phenotypes were included, but not all mothers had biochemically proven hyperandrogenism. However, 58.2% of women presented with hyperandrogenemia, and 84.8% with clinical hyperandrogenism. Moreover, PCOS women had significant higher androgen levels when compared to non-PCOS women. Nevertheless, further studies should investigate how different maternal PCOS phenotypes influence the hormonal levels of the offspring.

RIA and ELISA were used as the routine detection methodologies for determining androgen levels in our study, and we are aware of issues related to these methods. However, the measurement of fT via RIA is well established [61]. Nevertheless, our results have to be interpreted with caution, as we did not use a gold standard method such as mass spectrometry for measuring androgens. However, the methods were applied to all samples with the same technology and were not changed throughout the whole study period. For AMH levels the assay was changed in November 2014, as mentioned in the methods section.

## 5. Conclusions

We believe that our reported results are of importance for physicians dealing with pregnant women known to have PCOS and may help in counselling. Although a negative effect of the higher androgen concentrations in PCOS mothers during pregnancy cannot be ruled out, the fact that androgen levels in female and male offspring of PCOS and non-PCOS women do not differ at birth, can reassure the patients.

## Figures and Tables

**Table 1:** Comparison of demographics.

	PCOS Women *n* = 79		non-PCOS Women *n* = 354		
	Mean *n*	±SD %	Mean *n*	±SD %	*p*-Value
Maternal characteristics					
Maternal Age (years)	30.6	±4.6	30.3	±5.1	0.660
Body mass index (kg/m^2^)	29.8	±6.1	28.9	±5.0	0.241
Smoking	3	3.8%	31	8.8%	0.169
Gestational diabetes	12	15.2%	21	5.9%	0.009
Pregnancy-induced hypertension	8	3.8%	17	4.8%	0.103
Operative delivery	41	51.89%	148	41.8%	0.259
Neonatal characteristics					
Gestational age (days) at delivery	279	±9.6	281.2	±6.9	0.058
Large for gestational age (>90th percentile)	3	3.8%	19	5.37%	0.779
Small for gestational age (<10th percentile)	9	11.39%	44	12.43%	1.0

**Table 2:** Comparison of androgens and anti-Mullerian hormone (AMH) in PCOS women and non-PCOS women. SHBG: sexual hormone-binding globulin.

	PCOS Women *n* = 67/79		non-PCOS Women *n* = 316/354		*p*-Value
Testosterone (ng/mL)	1.17	0.44–4.23	0.97	0.18–5.56	<0.001
Fee testosterone (pg/mL)	6.79	1.37–26.8	6.72	0.48–27.9	0.563
Androstenedione (ng/mL)	3.44	1.06–10.0	2.74	0.49–10.0	0.002
AMH (ng/mL)	1.10	0.10–25.0	0.72	0.02–49.0	0.001
SHBG (nmol/L)	200.00	173.49–494.89	637.67	200.00–997.25	0.029

Data presented as median (range) and comparison by Mann-Whitney U-test.

**Table 3:** Comparison of androgens and AMH in PCOS girls, non-PCOS girls, PCOS boys, and non-PCOS boys.

	PCOS Girls *n* = 27/36		non-PCOS Girls *n* = 151/178		*p*-Value *	PCOS Boys *n* = 29/43		non-PCOS Boys *n* = 149/176		*p*-Value **	*p*-Value ***
Testosterone (ng/mL)	1.54	0.84–5.82	1.82	0.88–13.05	0.230	2.17	1.16–10.10	1.76	0.95–9.52	0.120	0.021
Fee testosterone (pg/mL)	22.66	10.52–47.0	23.89	6.12–72.01	0.196	29.94	6.39–61.41	24.08	5.23–73.07	0.094	0.012
Androstenedione (ng/mL)	2.19	1.08–7.77	2.78	0.83–8.06	0.113	3.47	126–7.93	2.92	0.83–9.24	0.039	0.018
AMH (ng/mL)	0.20	0.00–9.2	0.20	0.00–25.0	0.975	22.0	14.4–45.6	20.01	1.6–124.2	0.395	0.001
SHBG (nmol/L)	32	20.17–39.0	35	1.2–81.6	0.292	38.5	20.1–62.0	37.85	18.3–105.9	0.919	0.021

Data presented as median (range) and comparison by Mann-Whitney U-test; * = comparison between PCOS girls and non-PCOS girls; ** = comparison between PCOS boys and non-PCOS boys; *** = comparison between PCOS girls and PCOS boys.
